# Genomic and Gene-Level Distribution of Histone H3 Dimethyl Lysine-27 (H3K27me2) in Arabidopsis

**DOI:** 10.1371/journal.pone.0052855

**Published:** 2012-12-28

**Authors:** Sunchung Park, Sookyung Oh, Steve van Nocker

**Affiliations:** 1 Plant Research Laboratory, Michigan State University, East Lansing, Michigan, United States of America; 2 Department of Horticulture, Michigan State University, East Lansing, Michigan, United States of America; Rush University Medical Center, United States of America

## Abstract

Histone lysine methylation patterns underlie much of the functional diversity of nucleosomes in eukaryotes, and an interesting aspect of histone methylation is the potential functional specificity for different methylation states on a given lysine. Trimethylation of histone H3 (H3K27me3) is intimately related to developmental gene silencing through the so-called Polycomb Group (PcG) mechanism. How this modification becomes established at PcG-repressed loci is generally not known, but it has been suggested that it may be facilitated by prior occupancy by H3K27me2. In this study we mapped the genomic and gene-level distribution of H3K27me2 in *Arabidopsis thaliana* using ChIP and a high-density tiling microarray, and integrated this with previous maps of other chromatin features and gene expression data. At the genome level, H3K27me2 enrichment sites were sparsely distributed across chromosomes, within an average size expected for a single nucleosome, and contrasted with the longer domains seen for H3K27me3. In both heterochromatic and euchromatic segments of the genome, H3K27me2 enrichment was often localized within transposon-related genes, with the longest genomic stretches of this modification corresponding to retroelements. However, H3K27me2 was more frequently found within protein-coding genes. These genes generally also showed moderate enrichment for H3K27me3, but H3K27me2 was strongly depleted within those genes most enriched in H3K27me3. H3K27me2 within highly transcribed genes was at highest levels at transcriptional starts and was strongly depleted throughout the transcribed regions, and reached higher levels at active than at silent promoters.

## Introduction

In the nucleus of eukaryotic cells, DNA is packaged as a dynamic fabric called chromatin, involving complex architecture that profoundly affects recombination, DNA modification and repair, and transcription [1]–[5]. At its simplest level, chromatin takes the form of the nucleosome, in which DNA is wrapped around a histone core consisting of a tetramer of H3/H4 and two heterodimers of H2A/H2B. Structural diversity at the nucleosomal level can be imparted by the substitution of ‘variant’ histones for canonical H2A and H3 [6], and by various posttranslational modification of the histones, including acetylation, phosphorylation, ADP-ribosylation, sumoylation, ubiquitination, and methylation [7], [8]. Histone substitution and modification can affect interactions between histones within the nucleosome core, interactions between the histones and DNA, and interactions among neighboring nucleosomes. In addition, histone modifications influence recruitment of a diverse array of factors that act as effectors of chromatin function. The potential for combinatorial modifications to lead to distinct readouts of chromatin function has been termed the ‘histone code’ [7].

Arguably the most interesting of histone modifications, especially as it relates to transcription, are methylations of specific lysines (K4, K9, K27, and K36) within the amino-terminus of H3. These lysines are subject to mono-, di-, or tri-methylation, adding to structural and functional diversity. In yeast, plants and metazoans, methylation of lysines at positions 4 and 36 (H3K4 and H3K36) has been characterized as a signature of active genes. For example, nucleosomes containing tri-methyl H3K4 (H3K4me3) tend to localize near the transcriptional start site (TSS) of genes transcribed by RNA polymerase II (Pol II) [9]–[14], whereas H3K36me3-enriched nucleosomes accumulate within the transcribed region and 3′ ends of such genes [15], [16]. In contrast, in fission yeast, plants and metazoans, methylation at H3K9 is generally associated with constitutive heterochromatin, transcriptional silencing, and DNA methylation [17]–[19].

Methylation of H3K27 is an elaboration seen in multicellular eukaryotes. In the reference plant *Arabidopsis thaliana* (Arabidopsis), mono-methylated H3K27 (H3K27me1) is a feature associated with heterochromatic chromocenters [20], [21], which in plants comprise mainly the extensive rDNA loci and highly repetitive DNA within the pericentromeric regions [22]. Within this heterochromatin, the H3K27me1 mark is written by ATXR5 and ATXR6 [23], two of the large family of SET-domain proteins in Arabidopsis [24]. The activity of ATXR5 and ATXR6 is required for heterochromatin maintenance, potentially a function of their repression of inappropriate replication [25]. H3K27me1 may also be found in chromatin outside of chromocenters, albeit in lower abundance [23]. AtSUVH2 has also been reported to be required for H3K27me1 *in vivo* [26]. In contrast to H3K27me1, H3K27me3 in Arabidopsis is found in euchromatin [20], [21], [26], [27], where it marks weakly expressed and developmentally silenced genes [13], [28]. H3K27me3 is written by the E(z) - like methyltransferase components of the PRC2 protein [29], which in Arabidopsis include CURLY LEAF, SWINGER, and MEDEA, and translated to silencing through a mechanism involving interaction with the HP1 homolog, LHP1 [30]. Although mechanisms of silencing via PRC2 and H3K27 methylation are generally not described, it is clear that the trimethyl form of H3K27 is the ‘active’ form for silencing [30].

Much less is known about the genomic and gene-level distribution of the intermediate H3K27me2 form. Immunofluorescence data from two studies in Arabidopsis suggested that H3K27me2 was found predominately within heterochromatic chromocenters [20], [21], [26]. However, a more recent study, also based on immunostaining, suggested that this modification localizes mainly outside of chromocenters, dispersed in many discrete foci [23]. These differences could be explained by specificity of the antibody used, as well as the conditions used for detection. Using chromatin immunoprecipitation (ChIP), Schubert et al [31] found H3K27me2 in limited domains within broader H3K27me3-enriched regions at two genes subject to PcG repression, *SHOOTMERISTEMLESS* (*STM*) and *AGAMOUS (AG)*. When mapped by ChIP at low resolution across only Chromosome IV [32], H3K27me2 was found within both heterochromatin and euchromatin. In heterochromatin, it occurred in large domains, whereas within euchromatin, it was distributed among transposon-related and protein-coding genes [32].

An interesting question is the potential functional specificity for different methylation states of H3K27, and the degree to which these states might be interconverted, potentially as a regulatory mechanism of transcription [33], [34]. To gain insight into potential functions of H3K27me2 in transcription and the relationship between this modification and H3K27me3, we examined distribution of H3K27me2 in Arabidopsis utilizing ChIP and high-density tiling arrays covering the entire euchromatic portion of the Arabidopsis genome. These data were examined in the context of H3K27me3 measurements from the same samples and utilizing the same platform.

## Materials and Methods

### Sample preparation, chromatin immunoprecipitation and microarray hybridization

Arabidopsis [ecotype Columbia (Col)-0] plants were grown in soil under long-day (18 h light/6 h dark) photoperiod, and aerial tissues were collected from 14-d-old plants. Chromatin was prepared as previously descibed [13]. Antibody specific for H3K27me2 is documented by the manufacturer (Upstate; catalog no. 07-322). To demonstrate specificity of this antibody under the conditions used in this study, mono-, di-, and trimethylated H3K27 peptides (Millipore; catalog nos. 12–567, 12–566, and 12–565, respectively) were resolved on SDS-polyacrylamide gels, transferred to nitrocellulose membranes (Amersham), and subjected to hybridization using a 1∶2000 final concentration of antibody. Detection utilized horseradish peroxidase (HRP)-conjugated anti-rabbit IgG (Bio-Rad) in combination with chemiluminescence (Amersham ECL). ChIP was carried out as previously described [13]. Input DNA (pre-IP) was prepared from 10% of the sonicated chromatin extracts by performing cross-link reversal and DNA purification in parallel with IP samples. Each IP and input was replicated using biologically independent samples.

Microarray analysis employed the Affymetrix GeneChip Arabidopsis Tiling 1.0R Array containing probes at 35-bp resolution across ∼97% of the ∼135 Mbp Arabidopsis genome. IP and input DNAs from two biological samples were linearly amplified using random primers, fragmented by enzymatic digestion to ∼100 bp, and end-labeled with biotin as described in the Affymetrix Chromatin Immunoprecipitation Assay Protocol (P/N 702238). Hybridization and scanning of microarrays were performed at the Research Technology Support Facility at Michigan State University. Raw data from these experiments have been deposited in the NCBI Gene Expression Omnibus (GEO), accession number GSE7907.

### Data analysis

Signal intensities [perfect match (PM)-mismatch (MM)] from two independent biological replicates were quantile-normalized after log_2_-transformation using the TileMap package [35]. Subsequently, signals from IP and input DNA were linearly scaled to the same mean. A log ratio of the average IP to input value was calculated for each probe. The normalized signals were adjusted for total H3 as needed for further analyses as reported previously [13].

To select significantly enriched genomic regions for H3K27me2 relative to H3 content, probe-level *t*-statistics were computed for each probe, and then neighboring probes were combined by applying a hidden Markov model to the probe-level statistics with a maximal gap of 1000 bp, a minimal run of 200 bp and posterior probability cutoff of 0.5. All procedures were performed using TileMap package and custom Perl scripts. The identified genomic regions were filtered for annotated genes using annotation of the genome provided by The Arabidopsis Information Resource (TAIR), in which transposable element genes and pseudogenes are annotated separately (ftp://ftp.arabidopsis.org/home/tair/Genes). Definition of euchromatic or heterochromatic portions of the genome was as given by Bernatavichute et al [36] based on abundance of repeats, genes and DNA methylation.

To generate genic positional profiles for global patterning and clustering analyses, we included only those genes spaced 350 bp or greater from an adjacent gene at the 5′ end, and 150 bp or greater from an adjacent gene at the 3′ end, as described previously [13]. This subset contained 17,233 of the 33,003 annotated nuclear genes and included 13,576 protein-coding genes, 2,807 transposon-related genes, and 527 pseudogenes. Genic profiles were derived by analyzing probe signals for 100-bp windows within the proximal promoter (−350 bp to −50 bp relative to the TSS), TSS region (−49 bp to 0 bp to 5% of transcribed region), transcribed region (intervals of 10% of transcribed region from 5% to 95%), 3′ end region (from 95% to 100% of transcribed region to +50 bp relative to the 3′ end), and 3′ flanking region (51 bp to 150 bp relative to the 3′ end). For K-means clustering, we clustered multiple targets simultaneously by concatenating positional profiles of the selected genes for all target groups. Clusters were visualized with Treeview (rana.lbl.gov/EisenSoftware.htm). Gene expression levels and tissue-specificity (Shannon entropy) were estimated from archived transcriptional data as described previously [13] using AtGenExpress data sets 490, 491 and 492, corresponding to 21-, 22-, and 23-d-old whole plants, respectively.

## Results

### Genomic and gene-level distribution of H3K27me2

To map genomic and genic distribution of H3K27me2 at high resolution in Arabidopsis, we carried out chromatin immunoprecipitation (ChIP) followed by hybridization of immunoprecipitated DNAs to the Affymetrix GeneChip® Arabidopsis Tiling 1.0R microarray, as previously described [13]. For these experiments, we used aerial parts of whole plants, for which previous gene expression and chromatin-related data was available [13], and a commercial antibody generated against a synthetic peptide. This antibody has been used widely for conventional ChIP analysis in Arabidopsis [37]–[39], but its specificity has not been previously documented. We demonstrated the specificity of the anti-H3K27me2 antibodies for the dimethylated form of H3K27 under our conditions by western blotting of synthetic peptides (Fig. S1). Microarray hybridization signals were normalized to those from parallel hybridizations using input (non-immunoprecipitated) DNAs. Normalized signals were interpreted relative to total H3 signals derived using an antibody recognizing the conserved carboxyl-terminal domain of H3 irrespective of modification, as previously reported [13]. Importantly, this stringent control protocol eliminates artifacts stemming from nucleosome occupancy or microarray probe density. Correlation values (-R) of the two biological replicates showed high reproducibility (R≥0.879) (Fig. S2).

**Figure 1:**
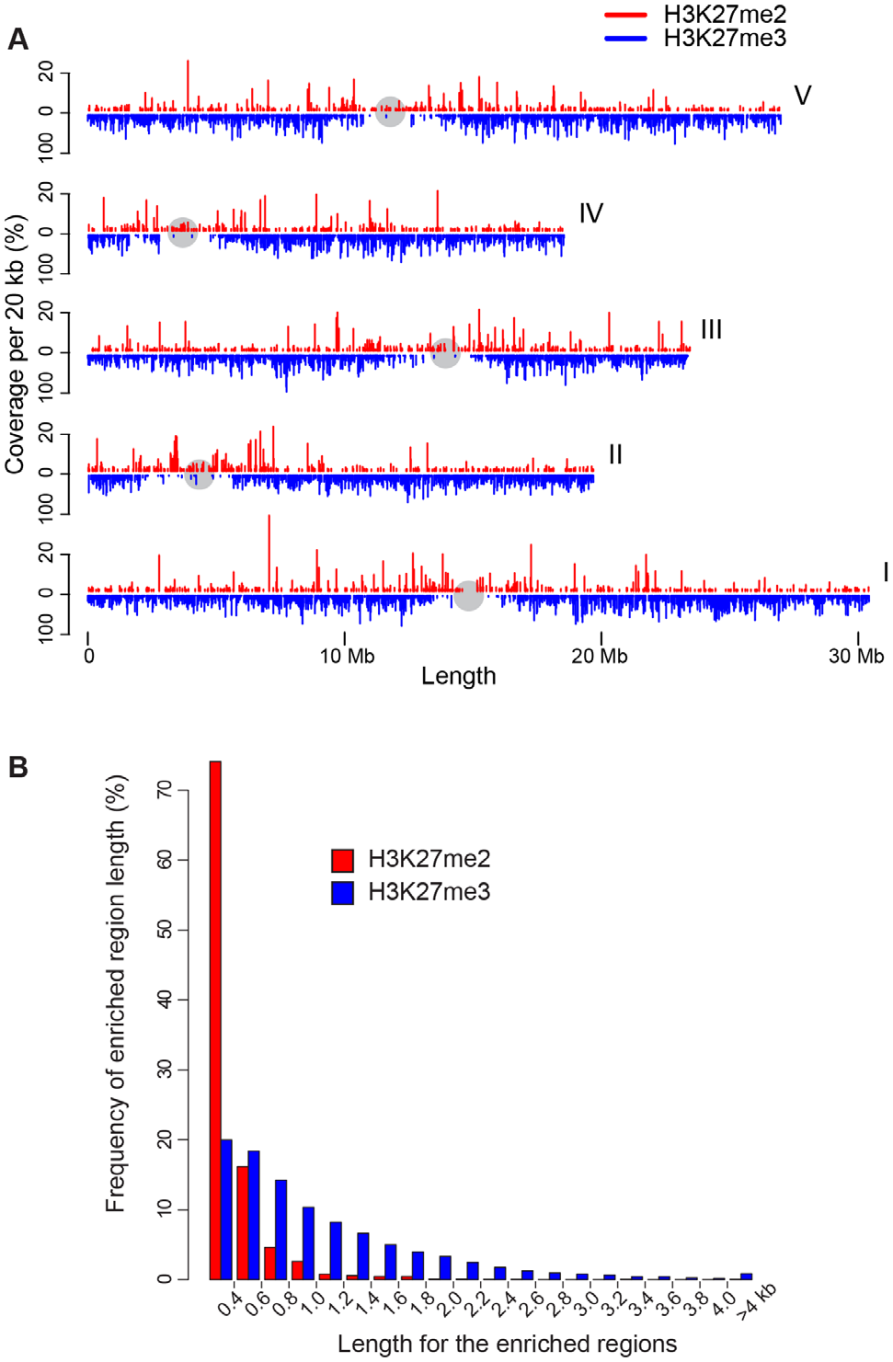
Chromosomal distribution and length of H3K27me2-enriched regions. (**A**) Chromosomal locations for the enriched regions for H3K27me2 (red) and H3K27me3 (indigo) are shown. The extent of the enriched regions across each of five chromosomes is represented as a function of % coverage per 20 Kbp, using different scales for H3K27me2 [0–20%] and H3K27me3 [0–100%]. The centromeric region of each chromosome is denoted with a gray sphere. (**B**) Graph of the size distribution of H3K27me2 peaks (red) and H3K27me3 peaks (indigo). The x axis indicates length of the enriched region, whereas the y axis indicates the percentage of the total number of enriched regions made up by each size class.

We determined genomic sites significantly enriched for H3K27me2 (enriched regions, or peaks), relative to H3 content, using a hidden Markov model based approach. H3K27me2-enriched regions were distributed across the breadth of chromosomes, including pericentromeric regions (Fig. 1A). However, a subtle increase in peak frequency could be seen near centromeres, especially for Chromosomes I and II. The highest frequency of H3K27me2-enriched regions was ∼1 Mbp proximal to the centromere of Chromosome II, corresponding to a region occupied predominately by a diverse array of transposon-related genes (data not shown). This genomic pattern was strikingly different than that previously observed for H3K27me3, which revealed occupancy for H3K27me3 within the gene-rich euchromatic chromosome arms, and marked depletion near centromeres [13], [28] (Fig. 1A). The combined extent of H3K27me2 peaks occupied a total of only 0.9 Mbp of the analyzed genome. This value was much smaller than that determined previously for H3K27me3 (18.2 Mbp) utilizing similar analysis parameters [13]. This relatively limited occupancy was a combined result of the enriched regions being both short and infrequent. For example, only 25% of H3K27me2-enriched regions were larger than 400 bp, compared with 80% for H3K27me3, and H3K27me2 was found in only 2,317 peaks compared with 13,470 for H3K27me3 (Fig. 1B and data not shown). Only ∼60 H3K27me2-enriched regions were greater than 1 Kbp in length. These long enriched regions represented ∼10% of the combined extent of peaks. Long enriched regions corresponded almost entirely to transposon-related genes, and of these, retroelements were strongly overrepresented, especially those of the LINE/L1 and LTR/Copia class. Overrepresentation of retroelements was highly significant even when differences in element length were considered (data not shown). For example, elements of the LINE/L1, LTR/Copia and LTR/Gypsy classes made up only ∼35% of all elements in the size range of ∼1–4 Kbp, but constituted nearly 90% of transposon-related genes within long H3K27me2-enriched regions (*P*<1E-40).

### H3K27me2 is commonly associated with transposon-related genes

**Figure 2:**
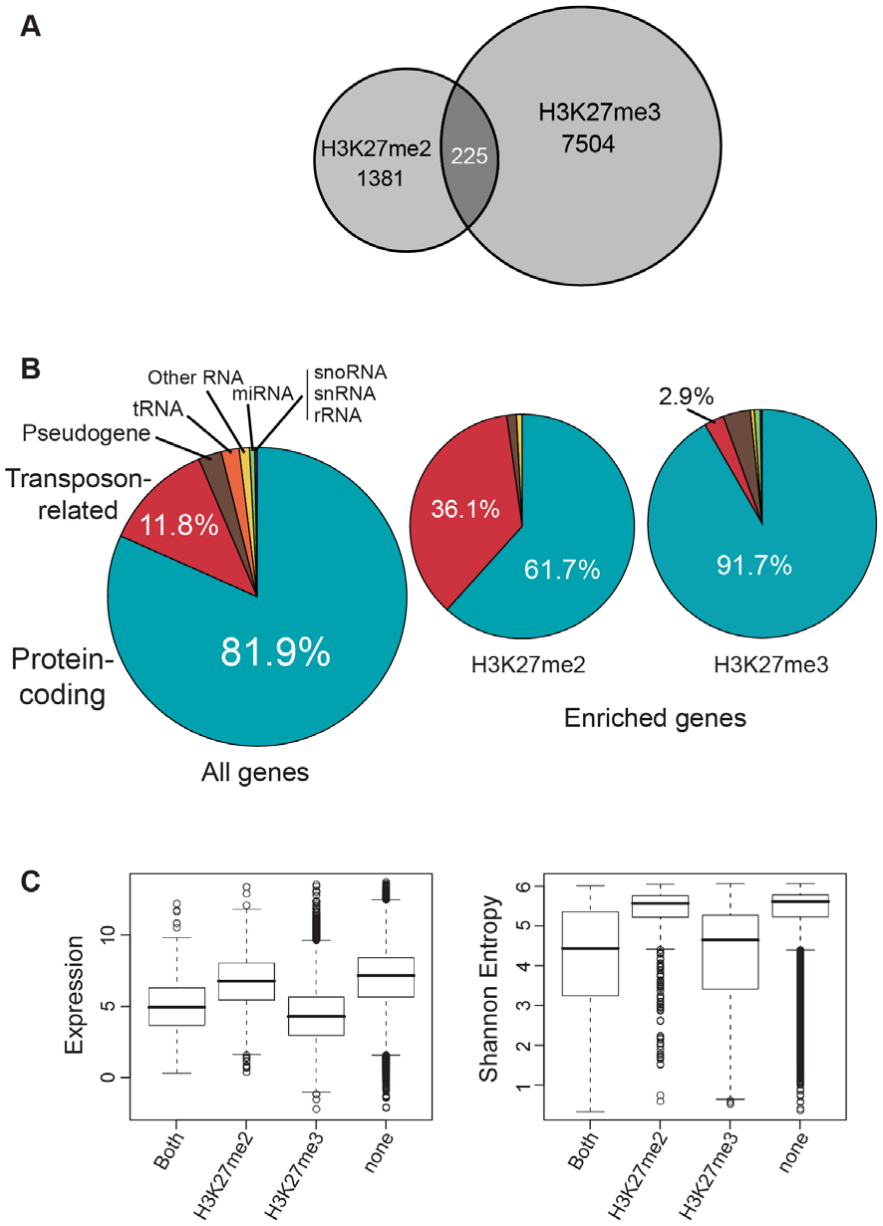
Classification of H3K27me2-enriched genes. (**A**) Venn diagram indicating the number of all annotated genes containing substantial enrichment for H3K27me2 and/or H3K27me3. (**B**) Pie charts indicating the representation of gene types for all annotated Arabidopsis genes, or for genes enriched in H3K27me2 or H3K27me3. For enriched genes, representation is specified with a percentage value for those gene types that are significantly over- or underrepresented. Additional data is shown in Table 1 and in Tables S1 and S2. (**C**) Box plots show estimated gene expression levels and tissue specificity for protein-coding genes enriched for H3K27me2, H3K27me3, or both, relative to genes not enriched for either. Tissue specificity is represented by Shannon entropy calculated from published microarray expression data [47], where lower values suggest more specificity [48]. Boxes indicate the 25th, 50th, and 75th percentiles (bottom, center line, and top of box, respectively).

To determine if enrichment for H3K27me2 was a general feature of transposon-related genes, we first utilized external annotation of the Arabidopsis genome to link H3K27me2 peaks with known genes. Genes were defined as enriched for H3K27me2 if any segment of the annotated transcribed region showed enrichment. Using this definition, ∼1,600 (∼5%) of the 33,033 annotated genes showed enrichment. This set was substantially smaller than the ∼7,700-gene set defined for H3K27me3 [13]. There was significantly less overlap between these sets than that predicted from random distribution (Chi-square *P*<0.001) (Fig. 2A and data not shown). We found that transposon-related genes were overrepresented among gene types associated with H3K27me2, whereas protein-coding genes were underrepresented, compared to representation of these gene types in the entire gene set (Fig. 2B and Table 1). No significant associations were found for other gene types, including pseudogenes or small RNA genes. The overrepresentation of transposon-related genes was highly significant (*P* value<1E-20; Fisher's exact test) within regions of the genome defined either as heterochromatin or euchromatin [36], showing that this phenomenon is not dependent on these generalized chromatin types (Table S1). This contrasts to the association of H3K27me3 with protein-coding genes rather than transposon-related genes [13], [28]. H3K27me2-enriched genes were collectively expressed at levels similar to genes lacking H3K27me2, and generally did not exhibit striking tissue-specific expression patterns (Fig. 2C), in contrast to H3K27me3-enriched genes, which are markedly weakly expressed and tissue-specific [13], [28].

**Table 1:** Gene types associated with H3K27me2- and H3K27me3-enriched regions.

Type	Entire (%)	No. of H3K27me2- enriched (%)	No. of H3K27me3- enriched (%)
Protein-coding	27025 (81.9)	991 (61.71)*	7090 (91.73)*
Transposon-related	3900 (11.8)	580 (36.11)*	225 (2.91)*
Pseudogene	859 (2.6)	24 (1.49)	287 (3.71)
tRNA	631 (1.9)	0 (0)	5 (0.06)*
Other RNA	326 (1)	11 (0.68)	54 (0.7)
miRNA	174 (0.5)	0 (0)	65 (0.84)
snoRNA	71 (0.2)	0 (0)	2 (0.03)
snRNA	13 (0.04)	0 (0)	1 (0.01)
rRNA	4 (0.01)	0 (0)	0 (0)

* Asterisk indicates that the proportion of the indicated gene type is significantly different from that in entire set of genes (Fisher's exact test P value<1E-40).

**Figure 3:**
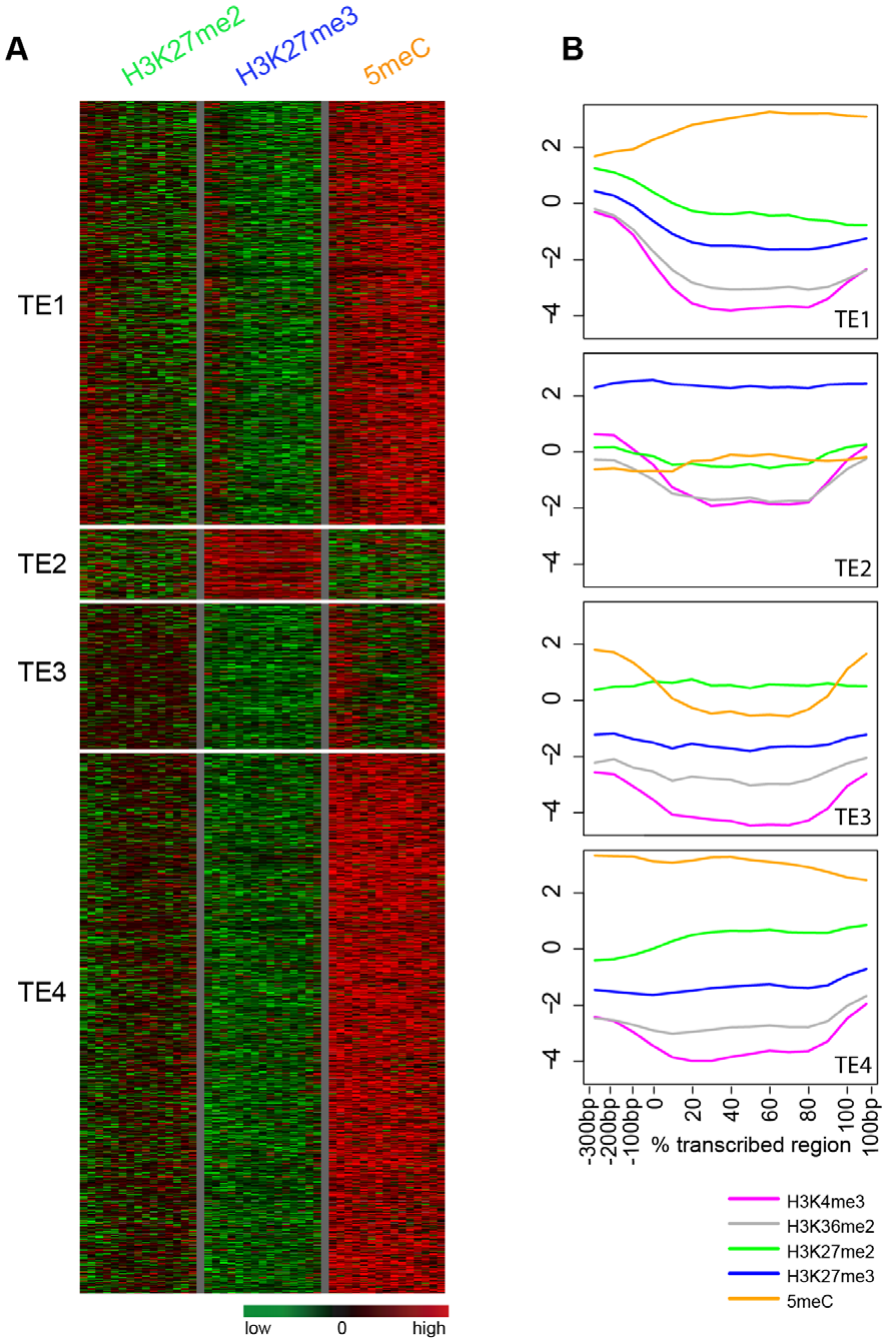
H3K27me2 and combinatorial modifications across transposon-related genes. (**A**) Cluster analyses were performed for transposon-related genes based on genic positional signals for H3K27me2, H3K27me3 and DNA methylation. Data was plotted across promoter regions (columns 1–3 in each modification panel), TSS (column 4), transcribed regions (columns 5–14) and 3′ end (column 15). (**B**) Averaged positional profiles for H3 modifications, including H3K4me3 and H3K36me2, and DNA methylation are shown for the resulting clusters. The y axis indicates the log2 of signal relative to H3.

We further investigated the relationship between H3K27me2 and H3K27me3 patterns within transposon-related genes. In this analysis, we also integrated published data for DNA cytosine methylation, which has been well characterized as associated with silenced tranposable elements [40]. Unsupervised K-means clustering of all transposon-related genes according to mean positional signals for H3K27me2, H3K27me3, and DNA methylation identified four general groups (Fig. 3). Three of these groups (Groups TE1, TE3 and TE4) showed H3K27me2 signals that were generally positive (relative to the genomic mean). Genes in Group TE1 showed strong H3K27me2 signals at the promoter and 5′ end, with decreasing levels throughout the gene body. Genes in Group TE3 showed relatively constant levels of H3K27me2 throughout the gene. Genes in Group TE4 showed weak H3K27me2 signals at the promoter and 5′ end, with increasing levels throughout the gene body. A distinguishing feature of genes in these three groups was high levels of DNA methylation (Fig. 3). In Groups TE1 and TE4, DNA methylation levels peaked at the 3′ end (TE1) or 5′ end (TE4), a pattern inversely related to that of H3K27me2. In contrast, in Group TE3, DNA methylation was found at both the 5′ and 3′ ends. Consistent with the known genomic distribution of DNA methylation, genes in all three of these H3K27me2-containing groups were located predominately in the pericentromeric regions of each chromosome (Fig. S3). The fourth K-means tranposon group, Group TE2, lacked marked H3K27me2 and DNA methylation, but was strongly marked by H3K27me3. Genes in this group also differed from other transposon-related genes in that they were not tightly clustered in the pericentromeric regions (Fig. S3). Genes in all four K-means groups were depleted for H3K4me3 and H3K36me2 (Fig. 3B), modifications that are generally localized within actively transcribed genes [13].

### H3K27me2 is found within protein-coding genes that contain moderate levels of H3K27me3

**Figure 4:**
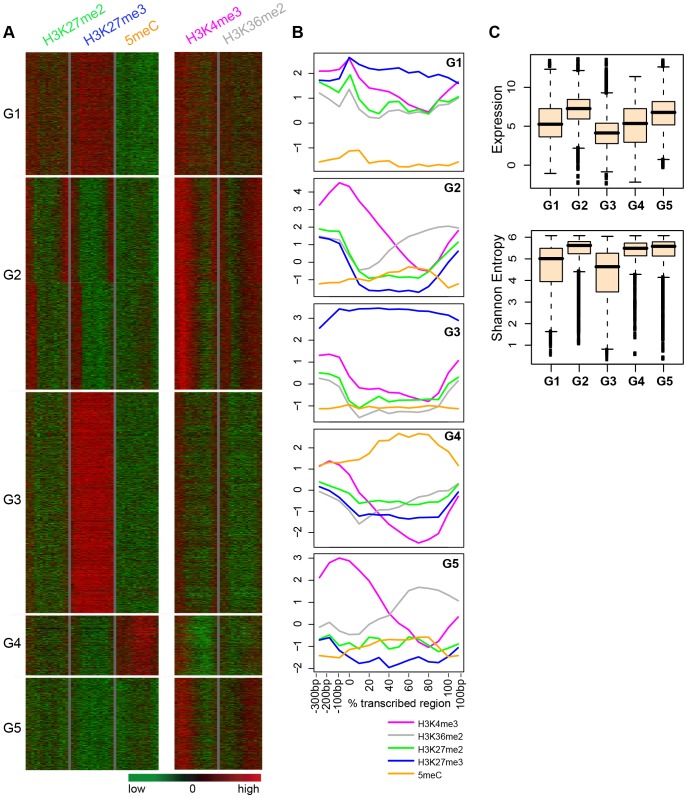
H3K27me2 and combinatorial modifications across protein-coding genes. (**A**) Cluster analyses were performed for protein-coding genes based on genic positional signals for H3K27me2, H3K27me3 and DNA methylation. The genic positional signals for H3K4me3 and H3K36me2 are also shown for the resulting five clusters. For each modification profile, data was plotted across promoter regions (columns 1–3 in each modification panel), TSS (column 4), transcribed regions (columns 5–14) and 3′ end (column 15). (**B**) Averaged positional profiles for H3 modifications and DNA methylation are shown separately for each of the five groups. The y axis indicates the log2 of signal relative to H3. (**C**) Box plots showing the level of expression (upper panel) and expression entropy (lower panel) for each group. Boxes indicate the 25th, 50th, and 75th percentiles (bottom, center line, and top of box, respectively).

K-means clustering of all protein-coding genes according to mean positional signals for H3K27me2, H3K27me3, and DNA methylation identified two distinct groups with generally positive H3K27me2 signals (Fig. 4A,B). Group 1 (G1) genes showed moderate signals for H3K27me2 across the entire transcribed region. G1 genes also showed moderate signals across the transcribed region for H3K27me3, H3K4me3, and H3K36me2, and tended to lack cytosine methylation. Such genes generally showed low to moderate expression, and moderate to high tissue-specificity (Fig. 4C). G1 genes also showed slightly higher expression and less tissue-specificity than genes with high signals only for H3K27me3 (G3). Group 2 (G2) consisted of genes characterized by strong H3K27me2 signals in the promoter and 3′ regions and depletion throughout the transcribed region. G2 genes also showed parallel pattern of signals for H3K27me3, a subtle peak of DNA methylation within the transcribed region and strong signals for H3K4me3 and H3K36me2 near the TSS and 3′ end, respectively. Such genes tended to be strongly expressed and to show a low degree of tissue specificity (Fig. 4C), and collectively were strongly underrepresented for function in ‘unknown biological processes’ (Table S2). Two additional groups defined by this analysis, G4 and G5, showed generally negative H3K27me2 signals, and high degree of DNA methylation (G4), or absence of both H3K27me3 and DNA methylation (G5). G5 genes tended to exhibit strong TSS/5′ signals for H3K4me3 and 3′ signals for H3K36me2 typical of strongly transcribed genes.

### H3K27me2 in protein-coding genes is enriched at 5′/3′ ends and depleted within transcribed regions

**Figure 5:**
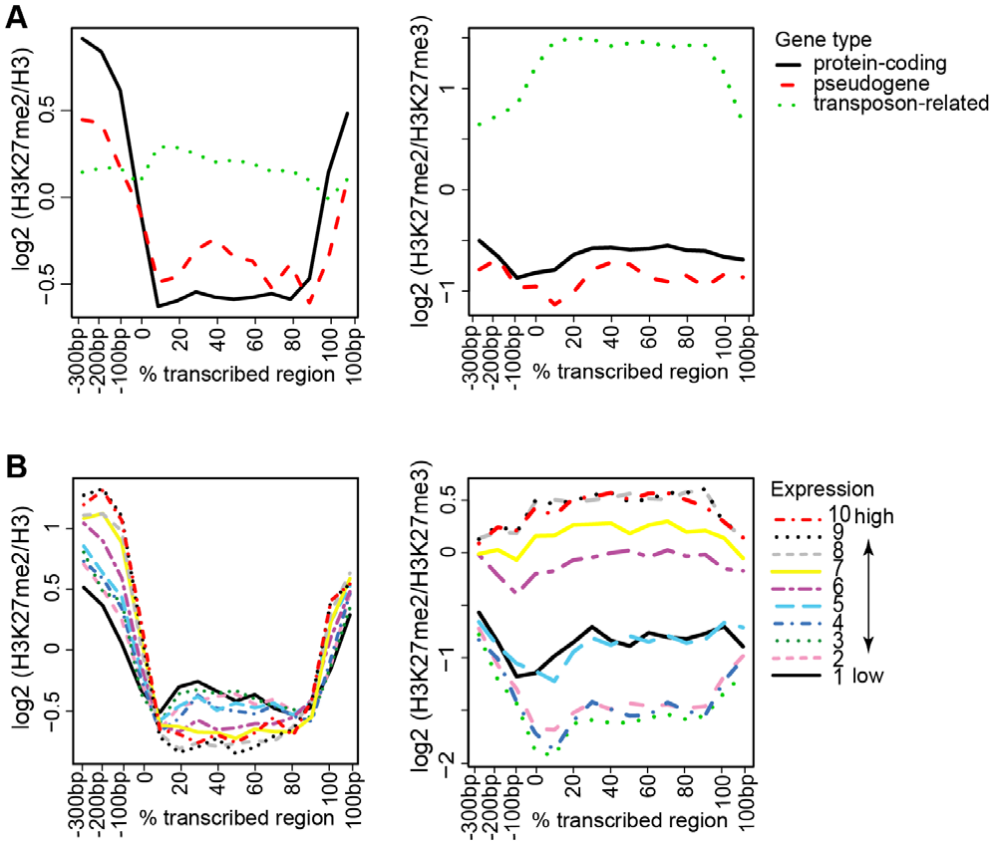
Levels of H3K27me2 across transcriptional units according to gene type or expression level. (**A, Upper panels**) Mean genic positional signals for H3K27me2 were calculated for 13,576 protein-coding genes, 527 pseudogenes, and 2,807 transposon-related genes, and is depicted across the promoter regions (shown in bp from −300 to 0 bp relative to the presumed transcriptional start site), transcribed regions (shown proportionally from 0 to 100% of total length), and 3′ regions (shown in bp from 0 to +100 relative to the presumed 3′ end). (**B, Lower panels**) Positional signals for protein-coding genes were sorted into ten-percentile bins according to expression level, as estimated from publicly available microarray data (see Materials and Methods). For (A) and (B), panels at right show H3K27me2 signal relative to that for H3K27me3 [13].

We calculated the mean levels of H3K27me2 at defined positions across the proximal promoter, transcribed region, and immediate 3′ flanking sequence for protein-coding genes, pseudogenes, and transposon-related genes. Protein-coding genes collectively showed moderate levels in the promoter/TSS/5′ and 3′ end, with depletion throughout the transcribed region (Fig. 5A). The pattern across pseudogenes resembled that of protein-coding genes, although with weaker signals for occupancy and depletion. In contrast, transposon-related genes tended to show relatively weak but even levels of H3K27me2 across the transcriptional unit (Fig. 5A). For protein-coding genes and pseudogenes, the genic H3K27me2 pattern mirrored that previously observed for H3K27me3 [13], which also showed relative levels at the promoter/TSS/5′ and 3′ ends and depletion throughout most of the transcribed region. For transposon-related genes, however, H3K27me2 was distinguished from H3K27me3 in that H3K27me3 showed lower levels within the transcribed region relative to promoter/TSS/5′ or 3′ regions [13]. Thus, transposon-related genes showed a higher H3K27me2/H3K27me3 ratio within the transcribed portion, relative to the promoter/TSS/5′ and 3′ region (Fig. 5A).

When gene activity was considered within the class of protein-coding genes, those genes that were the most strongly expressed showed the greatest relative levels at the promoter/TSS region and depletion for H3K27me2 across the transcribed region (Fig. 5B). This trend is similar to that reported previously for H3K27me3 [13], [28]. However, there is a clear distinction between these two marks; for H3K27me2, gene activity is associated with stronger signals at the promoter and stronger depletion within the transcribed region, whereas for H3K27me3, gene activity is associated with depletion throughout the gene [13]. Those genes in the top three deciles for expression level showed the highest H3K27me2 level relative to H3K27me3 at the promoter/TSS/5′ region (Fig. 5B).

## Discussion

Previous studies using immunostaining suggested H3K27me2 is preferentially localized to heterochromatic ‘chromocenters’ [20], [21], [26], whereas a more recent study concluded that H3K27me2 is localized outside of chromocenters, dispersed in many discrete foci, similar to the distribution of H3K27me3 [23]. Unlike the previous studies, Jacob et al [23] demonstrated specificity of the anti-H3K27me2 antibodies for the dimethyl form over the mono- or tri-methyl form. We demonstrated specificity of a distinct source of antibodies recognizing H3K27me2, and used ChIP combined with genomic tiling microarray analysis to characterize genomic- and gene-level distribution of H3K27me2. The finding that most H3K27me2 is distributed across chromosomes arms supports the observations of Jacob et al [23]. However, we also found H3K27me2 enriched domains within pericentromeric regions, where they were subtly increased in density, consistent with a recent analysis of H3K27me2 on Chromosome IV [32]. Jacob et al [23] suggested that apparent H3K27me2 detection in chromocenters might result from crossreactivity with H3K27me1. Although the antibodies that we used did not react with a synthetic H3K27me1 peptide in immunoblot analysis (Fig. S1), we cannot rule out minor crossreactivity of these antibodies under conditions used for ChIP. We did find that the genomic pattern observed here for H3K27me2 was strikingly different than that seen for H3K27me3, which was exclusively constrained to the gene-rich euchromatic chromosome arms (Fig. 1 and [13]). The pericentromeric signals for H3K27me2 may be explained by H3K27me2-enriched transposon-related genes, most of which were located near centromeres (Fig. S3).

Although mass-spectrometric analyses of histone H3 modifications has suggested that H3K27me2 is more abundant than H3K27me3 in Arabidopsis [41], we found that significantly enriched regions for H3K27me2 were both less common and shorter than those for H3K27me3 (Fig. 1). One explanation is that H3K27me2 could be widely dispersed throughout chromatin, but without statistically significant local concentrations identifiable as ‘enriched regions’ in this analysis. The median length of H3K27me2-enriched regions was approximately the size expected for a nucleosomal unit (<400 bp) (Fig. 1B). Thus, most ‘enriched regions’ reported here do not define multiple adjacent H3K27me2-modified nucleosomes, but instead probably define single nucleosomes modified with H3K27me2 across many or most cell types.

H3K27me2 domains marked a substantial portion of protein-coding genes, and such genes generally showed additional moderate enrichment for H3K27me3. However, H3K27me2 was strongly depleted within those genes with the highest levels of H3K27me3. This is consistent with the intuitive conversion of H3K27me2 to H3K27me3 associated with PcG repression. When analyzed through numerous cell types, as was done in this study, PcG-targeted genes that are repressed in the vast majority of the cell types would be expected to exhibit very strong signals for H3K27me3, but lack the H3K4me3/H3K36me2 signatures of transcription. This was seen in the G3 genes (Fig. 4). On the other hand, PcG-targeted genes that are repressed in some cell types, but active in others, would show lower, but still substantial, levels of H3K27me3, but additionally would show strong signals for H3K4me3/H3K36me2. This was seen in G1 and G2 genes (Fig. 4). The presence of substantial levels of H3K27me2 within G1 and G2 genes is consistent with a promotive mechanism of H3K27me2 for H3K27me3 accumulation. A priming mechanism for H3K27me3 requiring prior accumulation of H3K27me2 was previously hypothesized in metazoans by Sarma et al [34] based on observations of the PRC2 auxiliary subunit PHF1, which promotes H3K27me3 over H3K27me2. Such a mechanism was suggested in Arabidopsis by Schubert et al [31], who identified short H3K27me2-occupied regions within longer H3K27me3 domains at the PcG-targeted *AG* and *STM* genes. For *AG*, this included a regulatory element required for both activation and repression of the gene, and it was suggested that this might represent a PRE-like element. Short H3K27me2 domains would be expected to locally prime H3K27me3 formation by PRC2, which could spread across the transcriptional unit. If H3K27me2 domains are a general feature of PcG repression, then we might expect to find H3K27me2 domains within most or all larger domains enriched in H3K27me3. However, in this study we found that such domains were statistically excluded from regions most strongly enriched for H3K27me3. An obvious explanation is that, as described above, maximal occupancy by H3K27me3 that defines enriched regions is mutually exclusive with H3K27me2. Another function for H3K27 methylation may be to exclude H3K27 acetylation, which was shown to antagonize PcG silencing in flies [42] and in embryonic stem cells [43]. However, it is unclear whether such a mechanism might be employed in Arabidopsis, as acetyl-H3K27 was not detected through mass spectrometric analyses of bulk Arabidopsis histones [41], [44].

We found that higher gene activity is associated with stronger depletion of H3K27me2 within the transcribed regions of protein-coding genes, similar to observations for H3K27me3 [13]. This contrasts with the observations of Roudier et al [32] who found even distribution of H3K27me2 across transcribed regions. This distinction could be explained by the much higher resolution of microarrays used in our analysis (∼35 bp, compared with ∼900 bp), more comprehensive coverage of the genome (all euchromatic portions, compared with only Chromosome IV), and differences in the gene sets analyzed. Transcription-associated depletion could be accomplished by known mechanisms of H3/H4 exchange, in which ‘variant’ H3.2 (also H3.3) replaces ‘canonical’ H3.1 during histone eviction that accompanies passage of RNA polymerase II (RNAPII) [45]. Because H3.2/3 is apparently not modified by trimethylation at K27me3 and contains reduced dimethylation at K27 relative to H3.1 [41], this would lead to an apparently depletion after repeated rounds of transcription. An additional and unexpected finding was that H3K27me2 increased at the promoter/TSS/5′ ends associated with gene activity. This is perhaps surprising given the previous observation that levels of H3K27me3 at the promoter/TSS/5′ ends declines with gene activity [13], and could be explained by active conversion of H3K27me3 to H3K27me2 at transcriptional starts, perhaps by histone demethylases such as REF6 [46].

An outstanding question is how H3K27me2 becomes initially established in chromatin. We prefer a simple model where methylated H3K27 is released from transcribed regions during Pol II-associated nucleosome dissociation, and then becomes stochastically integrated, along with nascent histones, into new chromatin during DNA replication. Chromatin profiling of synchronized cell cultures may be effective to test this model. As exemplified in these discussions, analyses of chromatin profiles in whole organs is complicated by the view of chromatin through many cell types, in which profiles might be very distinct. Emerging technologies that allow chromatin profiling within isolated cell types should provide further resolution.

## Supporting Information

Table S1 **Gene types associated with H3K27me2-enriched regions throughout the genome and for euchromatic and heterochromatic regions.**
(PDF)

Table S2 **Functional categories of protein-coding gene clusters via Gene Ontology analysis.**
(PDF)

Figure S1 **Specificity of anti-H3K27me2 antibody.** Mono-, di-, or tri-methylated H3 (amino acids 21–44) peptide, as well as total Hela cell extract, were electrophoresed on an 18% SDS polyacrylamide gel and subjected to immunoblotting using anti-H3K27me2 antibody. The antibody reacted strongly with a single species of the predicted molecular mass (∼3 kDa) in the dimethylated H3K27 sample, and with a species of the molecular mass expected for H3 (∼17 kDa) in the Hela cell extract (right lane). An SDS-PAGE gel run in parallel and silver-stained is shown in the lower panel.
(PDF)

Figure S2 **Reproducibility of ChIP-on-Chip data.** An M versus A (MvA) plot representing signal intensities from the two biological replicates is shown for input (top), H3K27me2 (middle) and H3-CT (bottom). The x and y axes represent the average and difference, respectively, of the log base 2 of the intensities from the two replicates. The color bar at right indicates the number of probes on the plots.
(PDF)

Figure S3 **Chromosomal mapping of transposon-related gene groups.** Chromosomal locations for transposon-related genes are shown separately for each of the four clusters depicted in Fig. 3. The frequency of the transposon-related genes across each of five chromosomes is represented as a function of count per 20 Kbp. Protein-coding genes (H3K27me2-enriched Group 1 as shown in Fig. 4) are included as a general reference for euchromatic regions. The approximate position of the centromere is indicated with a gray box for each chromosome.
(PDF)
