# Supplementary material for: Genomic and Gene-Level Distribution of Histone H3 Dimethyl Lysine-27 (H3K27me2) in Arabidopsis
Source: PLoS One. 2012 Dec 28;7(12):e52855. doi: 10.1371/journal.pone.0052855 (PMC3532402; doi:10.1371/journal.pone.0052855)
Supplement: Table S1 — Gene types associated with H3K27me2-enriched regions throughout the genome and for euchromatic and heterochromatic regions. (PDF) [file pone.0052855.s001.pdf]

Table S1. Gene types associated with H3K27me2-enriched regions throughout the genome and for euchromatic and heterochromatic regions

Types	Euchromatic regions (%)		Heterochromatic regions (%)	
	Entire	H3K27me2-enriched	Entire	H3K27me2-enriched
Protein-coding	23892 (91.8)	890 (83.3)*	3133 (44.9)	101 (18.8)*
Transposon-related	577 (2.2)	157 (14.7)*	3323 (47.6)	423 (78.8)*
Pseudogene	474 (1.8)	13 (1.2)	385 (5.5)	11 (2)
tRNA	568 (2.2)	0 (0)	63 (0.9)	0 (0)
Other_RNA	277 (1.1)	9 (0.8)	49 (0.7)	2 (0.4)
miRNA	157 (0.6)	0 (0)	17 (0.2)	0 (0)
snoRNA	68 (0.3)	0 (0)	3 (0)	0 (0)
snRNA	13 (0)	0 (0)	0 (0)	0 (0)
rRNA	2 (0)	0 (0)	2 (0)	0 (0)

* Asterisks and colors indicate that the proportion of the indicated gene type is significantly different from that in entire set of genes (Fisher's exact test P value <1E-20); yellow for overrepresentation and green for underrepresentation relative to entire set.
