# Supplementary material for: Genomic and Gene-Level Distribution of Histone H3 Dimethyl Lysine-27 (H3K27me2) in Arabidopsis
Source: PLoS One. 2012 Dec 28;7(12):e52855. doi: 10.1371/journal.pone.0052855 (PMC3532402; doi:10.1371/journal.pone.0052855)
Supplement: Table S2 — Functional categories of protein-coding gene clusters via Gene Ontology analysis. (PDF) [file pone.0052855.s002.pdf]

Table S2. Functional categories of protein-coding gene clusters via Gene Ontology analysis

Biological process	Total (%)	G1	G2	G3	G4	G5
other cellular processes	7961 (23.6)	1098 (21.4)	3305 (26)*	1869 (21)*	461 (21.7)	1228 (25.6)
other metabolic processes	7056 (21)	1029 (20)	2662 (20.9)	1933 (21.7)	405 (19)	1027 (21.4)
unknown biological processes	4442 (13.2)	748 (14.5)	1125 (8.8)*	1474 (16.6)*	559 (26.3)*	536 (11.2)*
response to stress	1940 (5.8)	347 (6.7)	758 (6)	480 (5.4)	73 (3.4)*	282 (5.9)
protein metabolism	1889 (5.6)	169 (3.3)*	849 (6.7)*	361 (4.1)*	145 (6.8)	365 (7.6)*
developmental processes	1834 (5.4)	258 (5)	650 (5.1)	598 (6.7)*	105 (4.9)	223 (4.7)
response to abiotic or biotic stimulus	1799 (5.3)	333 (6.5)*	771 (6.1)*	413 (4.6)	55 (2.6)*	227 (4.7)
transport	1695 (5)	311 (6)	637 (5)	457 (5.1)	68 (3.2)*	222 (4.6)
other biological processes	1552 (4.6)	287 (5.6)	580 (4.6)	417 (4.7)	49 (2.3)*	219 (4.6)
transcription, DNA-dependent	1356 (4)	272 (5.3)*	427 (3.4)	490 (5.5)*	59 (2.8)	108 (2.3)*
signal transduction	893 (2.7)	142 (2.8)	359 (2.8)	203 (2.3)	53 (2.5)	136 (2.8)
cell organization and biogenesis	838 (2.5)	118 (2.3)	352 (2.8)	161 (1.8)*	71 (3.3)	136 (2.8)
DNA or RNA metabolism	294 (0.9)	20 (0.4)	169 (1.3)*	28 (0.3)*	20 (0.9)	57 (1.2)
electron transport or energy pathways	115 (0.3)	9 (0.2)	69 (0.5)*	7 (0.1)*	6 (0.3)	24 (0.5)

* Asterisks indicate that the proportion of the indicated gene type is significantly different from that in entire set of genes (Fisher's exact test P value <1E-05). Colors indicate those categories that were most significantly misrepresented (P<1E-20); yellow for overrepresentation and green for underrepresentation relative to entire set.
