# Supplementary figures and images for: Genomic and Gene-Level Distribution of Histone H3 Dimethyl Lysine-27 (H3K27me2) in Arabidopsis
Source: PLoS One. 2012 Dec 28;7(12):e52855. doi: 10.1371/journal.pone.0052855 (PMC3532402; doi:10.1371/journal.pone.0052855)

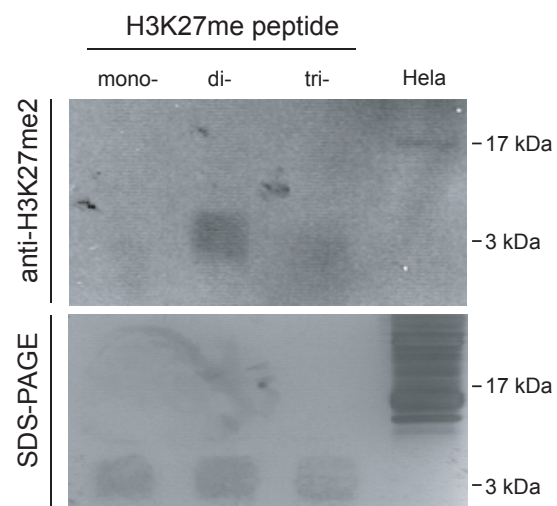

Supplement: Figure S1 — Specificity of anti-H3K27me2 antibody. Mono-, di-, or tri-methylated H3 (amino acids 21–44) peptide, as well as total Hela cell extract, were electrophoresed on an 18% SDS polyacrylamide gel and subjected to immunoblotting using anti-H3K27me2 antibody. The antibody reacted strongly with a single species of the predicted molecular mass (∼3 kDa) in the dimethylated H3K27 sample, and with a species of the molecular mass expected for H3 (∼17 kDa) in the Hela cell extract (right lane). An SDS-PAGE gel run in parallel and silver-stained is shown in the lower panel. (PDF) [file pone.0052855.s003.pdf]

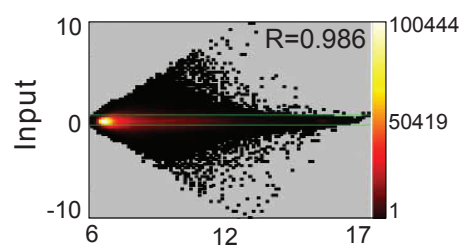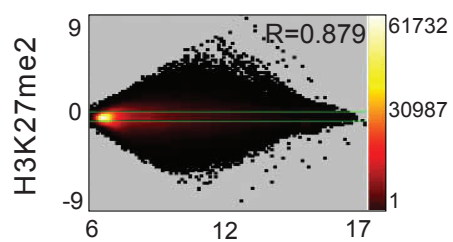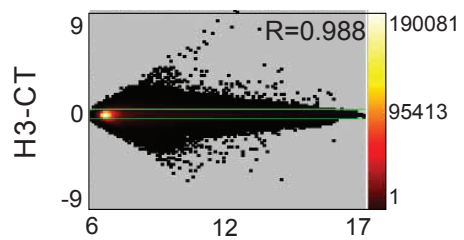

Supplement: Figure S2 — Reproducibility of ChIP-on-Chip data. An M versus A (MvA) plot representing signal intensities from the two biological replicates is shown for input (top), H3K27me2 (middle) and H3-CT (bottom). The x and y axes represent the average and difference, respectively, of the log base 2 of the intensities from the two replicates. The color bar at right indicates the number of probes on the plots. (PDF) [file pone.0052855.s004.pdf]

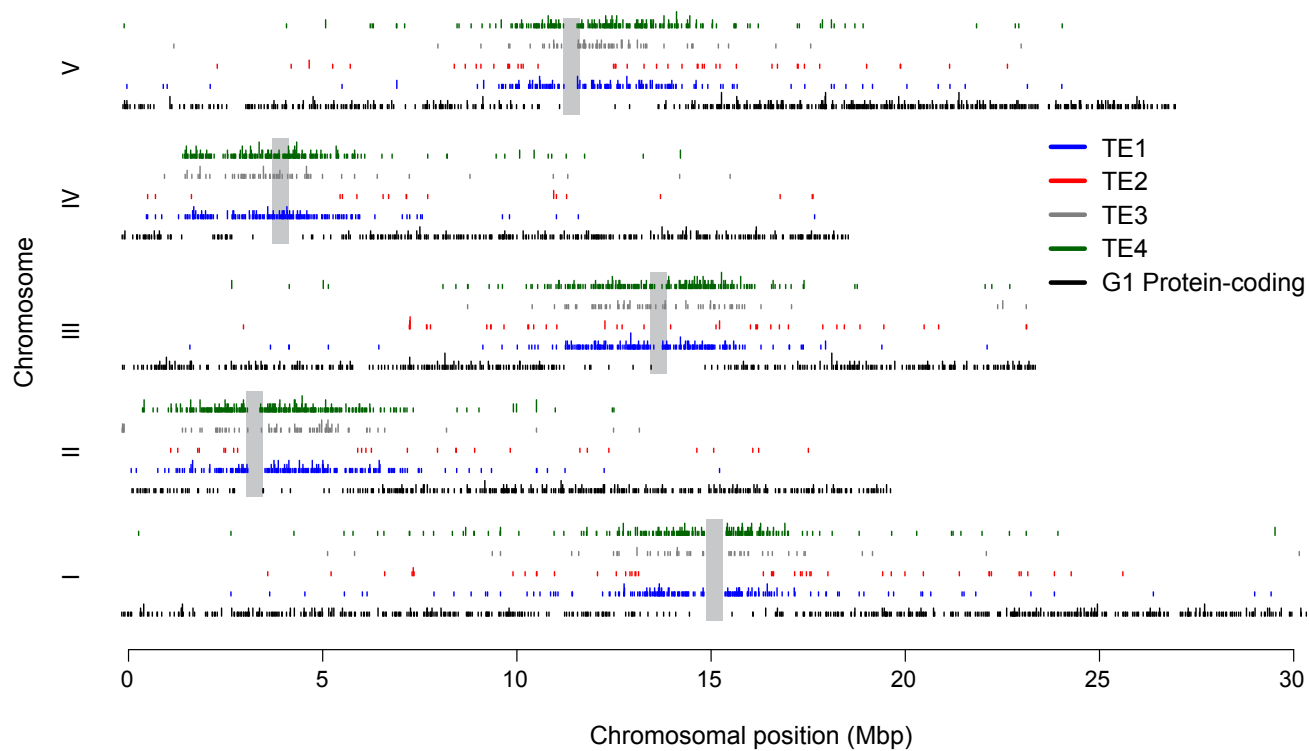

Supplement: Figure S3 — Chromosomal mapping of transposon-related gene groups. Chromosomal locations for transposon-related genes are shown separately for each of the four clusters depicted in Fig. 3. The frequency of the transposon-related genes across each of five chromosomes is represented as a function of count per 20 Kbp. Protein-coding genes (H3K27me2-enriched Group 1 as shown in Fig. 4) are included as a general reference for euchromatic regions. The approximate position of the centromere is indicated with a gray box for each chromosome. (PDF) [file pone.0052855.s005.pdf]
